# Evaluation of human monkeypox knowledge and beliefs regarding emerging viral infections among healthcare workers

**DOI:** 10.1186/s12245-023-00547-4

**Published:** 2023-10-18

**Authors:** Safa H. Alkalash, Marzouk M. Marzouk, Nagwa A. Farag, Fatma A. Elesrigy, Ayah M. Barakat, Faransa A. Ahmed, Rasha A. Mohamed, Abeer A. Almowafy

**Affiliations:** 1 Department of Community Medicine and Healthcare, Faculty of Medicine, Umm Al-Qura University, Al-Qunfudah, Kingdom of Saudi Arabia; 2 Family Medicine Department, Faculty of Medicine, Menoufia University, Menoufia, Egypt; 3 Department of Public Health and Community Medicine, Damietta Faculty of Medicine, Al-Azhar University, Damietta, Egypt; 4 College of Applied Medical Sciences in Alnamas, University of Bisha, Bisha, Kingdom of Saudi Arabia; 5 Pediatric Nursing, Faculty of Nursing, Assiut University, Assiut, Egypt; 6 Community Health Nursing Department, Faculty of Nursing, Mansoura University, Mansoura, Egypt; 7 College of Applied Medical Sciences, University of Bisha, Bisha, Kingdom of Saudi Arabia; 8 International Islamic Center for Population Studies and Research, Al-Azhar University, Cairo, Egypt

**Keywords:** Monkeypox, (HMPX), Orthopoxviruses, (HCWs), Biological warfare

## Abstract

**Objectives:**

The purpose of this study was to evaluate possible factors that might be accompanied by high level of human monkey pox (HMPX) knowledge and to explain the relationship between HMPX knowledge and Beliefs regarding emerging viral infections.

**Study design:**

A descriptive cross-sectional study was conducted for the implementation of this study.

**Methods:**

Study was conducted at two general hospitals in Mansoura City (Old General Hospital and International Hospital) El Dakahlia Governorate among 620 healthcare workers (HCWs) using a self-managed questionnaire for 1 week (1 to 7 January 2023). The questionnaire has items adapted from the previously published literature to assess HMPX knowledge and Beliefs regarding emerging viral infections.

**Results:**

The mean age of the study sample was 27.97 years and most of them were female (86.1%). Physicians and other HCWs (nurses, laboratory technicians, radiographer technicians, and pharmacists) had significantly different levels of knowledge of monkeypox for the majority of the questions. A higher belief was found among two items: viruses are biological weapons manufactured by the superpowers to take global control and the government is misleading the public about the cause of the virus.

**Conclusion:**

This study discovered lower levels of knowledge of HMPX among HCWs in Egypt. Beliefs about emerging viral infections were widespread, and future research should look into their potential negative impact on health behavior.

## Introduction

Monkeypox is a viral disease transmitted to humans from animals (zoonosis) due to MPXV of the Orthopoxvirus genus; this Genus comprises three other species pathogenic to humans [1]: cowpox virus, vaccinia virus, variola virus which causes smallpox [2]. In the late 1970s, smallpox was successfully eradicated from population [3]. MPXV was discovered in 1958 during an outbreak among monkeys in a Danish laboratory [4]. However, it stayed not recognized as a human disease until 1970, when a 9-month-old child in the Democratic Republic of the Congo (DRC), formerly known as Zaïre, became infected. In the tropical rain forests of the Congo basin (CB) and West Africa (WA) MPX is usually found [5–7], and DRC remains to report the majority of cases every year [8, 9], primarily in children under the age of ten. In the Central African Republic (CAR), the most recent epidemic occurred in October 2016, resulting in 26 cases, of which three were laboratory-confirmed. In 2003, the United States of America reported the first MPX epidemic outside of Africa (USA) (https://www.who.int/news-room/fact-sheets/detail/monkeypox), (after 800 small African mammals were shipped from Ghana into Texas [4]. HMPX will consider a potential threat in 2022 with increased cases in non-endemic regions [10]. The WHO declared on July 23 2022 that the monkeypox epidemic is a public health emergency of international concern. The Egyptian Ministry of Health and Population has reported 13 unconfirmed cases, of which only two have been confirmed. Patients have been quarantined in an isolation-designated hospital. With the patient’s contacts, all essential health and preventative measures have been taken in accordance with the treatment and follow-up protocols recommended by the World Health Organization (WHO). MPX virus is divided into two clades: West African and CB (Central African). CB clade appears to cause severe disease more frequently, with case fatality ratios (CFR) of up to 10% previously reported [11]. DRC has a CFR of around 3% among suspected cases. In an African population that is usually younger, the West African clade has previously been associated with a lower overall CFR of approximately 1%. There have only been a few deaths from MPX since 2017 [12]. HMPX is primarily transmitted through the saliva/respiratory tract or contact with infected animals’ skin lesions [13, 14]. High prevalence of cases among homosexual men and those having multiple sexual partners. The current HMPX outbreak raises concerns about the possibility of sexual transmission [15]. The disease’s clinical manifestation is similar to smallpox but less severe [16]. Fever, headache, back pain, myalgia, lymphadenopathy, and skin rash between the symptoms [17]. Cutaneous lesions, which progress from maculopapular to vesicles, pustules, and crusts, are most commonly present on the extremities and, in severe cases can occur anywhere on the body [16]. Possible consequences include secondary bacterial infections, respiratory problems, bronchopneumonia, gastrointestinal involvement, dehydration, sepsis, encephalitis, and corneal infections with subsequent vision loss. Since the MPXV infection is not currently treatable, patients are managed with supportive care and symptomatic therapy [18]. The main difference between smallpox and MPX is that the latter results in lymphadenopathy. HMPX prophylactic is based on the smallpox vaccine, which has been found to provide 85% protection [19]. The disease can persist for up to 4 weeks before the skin lesions disappear. In recent years, infectious illness outbreaks were frequently accompanied by the viral spread of misinformation, social media panic, and strange theories that could spread more quickly than the disease itself. theories are prevalent due to recent Ebola outbreaks and the coronavirus disease 2019 (COVID-19) pandemic [10]. Thus, it may be suggested that the extreme spread of theories surrounding the 2022 HMPX outbreak was to be expected. Despite the widespread acceptance of conspiracies appearing to be harmless, reports of potential negative effects have been made, especially in the circumstance of health-seeking behavior expressed in vaccine hesitancy and mistrust of scientific and medical organizations. Therefore, taking into account their significance in reducing the harmful effects of these beliefs, it is essential to investigate the prevalence of stage theories, especially among HCWs [20].

HCWs are an essential group taken into consideration for focused awareness and knowledge to be ready for providing the appropriate reactions, particularly during outbreaks and the emergence of infectious diseases. strang theories appeared during the current HMPX outbreak, including suspicions that the virus was bioengineered for a political reason. The primary responsibility of HCWs is to detect cases for early isolation and to immunize close contacts for control and prevention [21, 22].

Several studies have been carried out to evaluate the general public and HCWs’ knowledge, attitudes, and practices (KAP). Unsatisfactory knowledge (33.3%) of HMPX was discovered by Sallam et al. in their study [22] among HCWs. Additionally, according to Alshahrani, Najim Z., et al. about 55% of the survey respondents were found to have “good knowledge” of human MPX [23]. Another study of Saudi medical students revealed that 72% of them had inadequate knowledge of MPXV [24]. Thus, it is important to evaluate possible factors that might be accompanied by higher knowledge of HMPX and estimate the effects of such theories, particularly on the behavior of those who seek out health care.

## Method design of the subject

### Design

“The current cross-sectional study was based on the distribution of an online self-administered questionnaire to evaluate HMPX knowledge and Beliefs Regarding Emerging Viral Infections among Healthcare Workers. The occupational categories that fit our definition of HCWs included: physicians, nurses, pharmacists, and medical technicians”

### Setting

This research was carried out at two general hospitals in Mansoura City “Old General Hospital and International Hospital”, El Dakahlia Governorate for 1 week (1 to 7 January 2023).

### Sample

The study sample was collected using a convenience sampling technique. G-Power statistical software (version 3.1.9.7; Heinrich-Heine-Universität Düsseldorf, Düsseldorf, Germany) was used to calculate sample size [25]. When 620 participants were involved in the research, the power analysis revealed that an 80% power would be found with 95 percent confidence.

The inclusion criteria were as follows:

1. A healthcare worker practicing in the hospital during the epidemic.
2. Expressing readiness to answer a questionnaire. HCWs who participated in the study were provided with electronic informed consent and those who refused to answer a questionnaire were excluded.

### Study tools

The study researchers established a self-managed electronic questionnaire. Items in the questionnaire adapted from previously published literature were used to assess HMPX knowledge and strange theories about emerging viral infections.

The survey link of the electronic questionnaire “was created on Google Forms and shared on social media platforms like Facebook, Twitter, Instagram, Whatsapp, Telegram and the participants’ emails”. Responses to all items are mandatory to look.

The survey was divided into three parts:

I: Personal data
  Demographic data consisted of HCWs’ age, sex, workplace type, education, and profession.
II: HCWs’ knowledge of HMPX
  It was used to ask HCWs’ knowledge of HMPX and involved 13 questions. Questionnaire items were drawn from Harapan et al. [26]. The answers to each item were “yes” or “no”. Correct answers were scored as 1 and incorrect responses were scored as 0. The total scores were calculated for each item by adding the items’ scores (possible range of 0–13 marks). The frequency of each response selected and the percentages will be used to calculate the results (MPX K-score). Additionally, the mean score was calculated. Using a modified Bloom’s criteria cut-off point, the general knowledge scores of HCWs were classified as good if they were between 80 and 100% (10.4–13), moderate if they were between 60 and 79% (10.3–7.8), and poor if they were below 60% (total score 7.8). Knowledge about the monkeypox was evaluated using the following 13 items [27]:
  1. There is an outbreak of human MPX in the world.
  2. MPX is prevalent in Egypt.
  3. MPX is prevalent in Central and Western Africa.
  4. A virus causes MPX.
  5. Human-to-human transmission of MPX occurs easily.
  6. Smallpox and MPX have the same symptoms and signs.
  7. Skin rash is one of the symptoms or signs of HMPX.
  8. Vesicle is one of the symptoms or signs of HMPX.
  9. MPX could be transmitted through a bite from an infected monkey.
  10. A flu-like syndrome is one of the early signs or symptoms of humans.
  11. Antibiotics are utilized to treat HMPX.
  12. Diarrhoea is one of the symptoms or signs of HMPX.
  13. Vaccination is available to prevent HMPX.
III: Beliefs regarding viral infections:
  The items were adopted with the help of Freeman et al. studies on COVID-19 Medicine a 2020 beliefs [21, 22]. Ten-item questionnaires were used for the evaluation, and the potential answers were strongly disagree (1), disagree (2), neutral/have no opinion (3), agree (4), and highly agree (5) on a 5-Likert scale.
  The following items of beliefs:
  1. I am skeptical about the official explanation regarding the cause of virus emergence
  2. The virus is a hoax.
  3. Most viruses are artificial.
  4. I do not trust the information about viruses from scientific experts.
  5. The spread of viruses is a deliberate effort by a group of influential people to make money.
  6. The spread of viruses is a deliberate attempt by global companies to take control.
  7. Lockdowns in response to emerging infection are aimed at mass surveillance and to destabilize the economy for financial gain
  8. Lockdowns in response to emerging infections are aimed at mass surveillance and controlling every aspect of our lives.
  9. Lockdown is a way to terrify, isolate, and demoralize a society as a whole to reshape society to fit specific interests.
  10. Viruses are biological weapons manufactured by superpowers to take global control. Higher beliefs item scores showed a greater belief in Beliefs underlying the emergence of viruses and subsequent measures.

### Collection of data

The HCWs’ informed electronic consent to participate in the study was obtained prior to enrollment once the study’s objective had been clarified. The HCWs were informed that any topic may be clarified by researchers and personal relationships. A review of local and global literature was conducted on various aspects of HMPX using published scientific papers, and textbooks. After reviewing related literature, the study tools were advanced. To determine the extent to which the instrument measures what was hypothetical, a panel of five experts in community health nursing, epidemiology, and statistics accepted the devices.

The test-retest technique was employed to maintain score stability over a short period of time. The degree to which the survey items measure the same models was determined by measuring internal consistency. Internal stability was determined to be reliable by Cronbach’s alpha coefficient test to be (0.81). A pilot research on 10% of HCWs was presented (62 HCWs). They took great effort to measure the study tool’s clarity and applicability, divided the time needed for data collection, and identified any challenges or issues that might arise during data collection as well as any activities that might overload them. The necessary changes were made in response to the data that was collected, some questions were complemented and others were explained or absent.

### Statistical analysis

All the essential information was acquired at once and checked for extensiveness; using IBM SPSS for Windows software version 25 (IBM Corporation, Armonk, New York, NY, USA), they were coded, verified, and analyzed [28, 29]. With the use of calculated frequencies and proportions for qualitative data, the mean and standard deviation for quantitative data, statistics were developed to simplify data. Comparing qualitative data among physicians and other healthcare professionals was done using the chi-square test. Using logistic regression analysis, factors that are associated with more beliefs were identified. *P* values below 0.05 were regarded as statistically significant in this investigation.

### Ethical consideration

Ethical approval was obtained from the ethical committee at the International Islamic Center for Population Studies and Research, Al-Azhar University (IRB no.: 49/2022), about the study’s purpose and their participation was entirely voluntary and not-for-profit. The participants were made aware that there would be no consequences if they decided to leave the study at any time. The questionnaire’s first page contained a representation of electronic informed consent. The researchers created and saved their code numbers. The study ensured data confidentiality by keeping participants’ data anonymous.

## Results

### Characteristics of the study participants

**Fig. 1:**
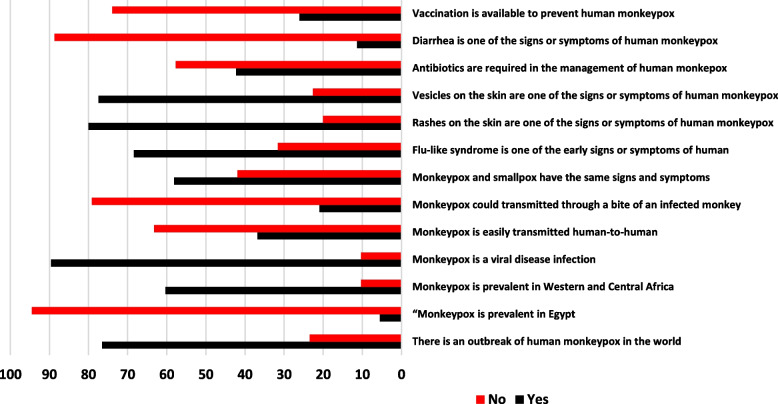
HCWS knowledge toward HMPX

The whole number of study participants was 620 HCWs. The common characteristics of the study respondents are demonstrated in Table 1. The mean age of the participants was 27.97 ± 7.32 years, the majority of the models were female (86.1%), and (69.4%) were characterized by university education and below. In addition, (65.5%) of the participants are working in community health care, and (84.5%) did not receive human monkeypox information during education. Additionally, (88.1%) did not hear about human monkeypox before. Figure 1 demonstrates the results of the HCWs’ knowledge of human monkeypox, the whole level of knowledge regarding HMPX was poor, with only four items having right response levels > 70%.

**Table 1:** Characteristics of the participants (*n* = 620)

**HCWs’ demographic characteristics**	***N* = 620**	**%**
**Age (years)**	Mean ± SD	
	27.97 ± 7.23	
30	594	95.8
≥ 30	26	4.2
**Gender**		
Female	534	86.1
Male	86	13.8
**Education**		
University and below	430	69.4
Postgraduate	190	30.6
**Profession**		
Physician	496	80
Other health care worker	124	20
**Type of workplace**		
Community health care	406	65.5
General hospital	124	20.1
Private hospital	90	14.4
**Have you ever received information about human monkeypox during education?**		
Yes	96	15.4
No	524	84.5
**Have you ever heard about human monkeypox before?**		
Yes	74	11.9
No	546	88.1

### Human monkeypox knowledge is divided by gender

A majority of the items (10/13) between males and females revealed no statistically significant variations in the degree of monkeypox knowledge, as compared to the three items in Table 2 where females demonstrated a significantly higher level of knowledge.

**Table 2:** Gender effect on human monkeypox knowledge

**Knowledge items**	**Gender**		***P* value**
	**Female *N* (%)**	**Male *N* (%)**	
**There is an outbreak of HMPX in the world**			
Yes	260 (83.8)	50 (16.2)	*P* = 0.454
No	274 (88.4)	36 (11.6)	χ2 = 4.123
**Monkeypox is prevalent in Egypt**			
Yes	8 (23.5)	26 (76.5)	*P* = 0.764
No	526 (89.7)	60 (10.3)	χ2 = 0.0929
**MPX is prevalent in Central and Western Africa**			
Yes	318 (86.9)	48 (13.1)	*P* = 0.001*,
No	216 (85.1)	38 (14.9)	χ2 = 19.05
**Monkeypox is a viral disease infection**			
Yes	382 (85.3)	66 (14.7)	*P* = 0.364
No	152 (88.4)	20 (11.6)	χ2 = 0.0619
**Monkeypox is easily transmitted human-to-human**			
Yes	196 (87.5)	28 (12.5)	*P* = 0.104
No	338 (85.4)	58 (14.6)	χ2 = 6.124
**MPX could be transmitted through a bite of an infected monk**			
Yes	108 (83.1)	22 (16.9)	*P* = 0.004*
No	426 (86.9)	64 (13.1)	χ2 = 14.124
**Smallpox and MPX and have the same symptoms and signs**			
Yes	314 (86.3)	50 (13.7)	*P* = 0.179
No	220 (85.9)	36 (14.1)	χ2 = 4.834
**The flu-like syndrome is one of the early symptoms or signs of human**			
Yes	358 (84.4)	66 (15.6)	*P* = 0.268
No	176 (89.8)	20 (10.2)	χ2 = 2.232
**Skin rashes are one of the signs or symptoms of HMPX**			
Yes	408 (86.4)	64 (13.6)	*P* = 0.321
No	126 (85.1)	22 (14.9)	χ2 = 4.544
**Blisters on the skin are one of the symptoms or signs of human monkeypox**			
Yes	386 (84.6)	70 (15.4)	*P* = 0.563
No	148 (90.2)	16 (9.8)	χ2 = 2.569
**Antibiotics are used in the treatment of HMPX**			
Yes	232 (86.6)	36 (13.4)	*P* = 0.435
No	302 (85.8)	50 (14.2)	χ2 = 0.943
**Diarrhea is one of the symptoms or signs of HMPX**			
Yes	106 (88.3)	14 (11.7)	*P* = 0.038*
No	428 (85.6)	72 (14.4)	χ2 = 8.627
**Vaccination is available to prevent human monkeypox**			
Yes	142 (86.6)	22 (13.4)	*P* = 0.354
No	392 (85.9)	64 (14.1)	χ2 = 2.748

*p < 0.05

### Human monkeypox knowledge is divided by professions

Physicians had a greater level of knowledge in eight items. On the other hand, no statistically significant variations were observed in the level of monkeypox knowledge between physicians and other HCWs demonstrated in Table 3.

**Table 3:** The level of HMPX knowledge between the study participants by professions

**Knowledge items**	**Professions**		***P* value**
	**Physician *N* (%)**	**Other HCWs *N* (%)**	
**There is an outbreak of human monkeypox in the world**			
Yes	264 (83.1)	54 (16.9)	*P* = 0.123
No	232 (76.8)	70 (23.2)	χ2 = 5.156
**Monkey pox is prevalent in Egypt**			
Yes	256 (79.5)	66 (20.5)	*P* = 0.118,
No	240 (80.5)	58 (19.5)	χ2 = 4.348
**MPX is prevalent in Central and Western Africa**			
Yes	278 (75.9)	88 (24.1)	*P* = 0.05*
No	218 (85.8)	36 (14.2)	χ2 = 8.697
**MPX is a viral disease infection**			
Yes	364 (78.1)	102 (21.9)	*P* = 0.003*
No	132 (85.7)	22 (14.3)	χ2 = 13.891
**MPX is easily transmitted human-to-human**			
Yes	188 (81.7)	42 (18.3)	*P* = 0.534
No	308 (78.9)	82 (21.1)	χ2 = 2.879
**MPX could be transmitted through a bite of an infected monk**			
Yes	90 (56.3)	70 (43.7)	*P* = 0.002*
No	406 (88.3)	54 (11.7)	χ2 = 12.354
**Smallpox and MPX have the same signs and symptoms**			
Yes	258 (73.3)	94 (26.7)	*P* < 0.001*
No	238 (88.8)	30 (11.2)	χ2 = 5.834
**The flu-like syndrome is one of the early symptoms or signs of human**			
Yes	388 (79.2)	102 (20.8)	*P* = 0.168
No	108 (83.1)	22 (16.9)	χ2 = 3.475
**Skin rashes are one of the signs or symptoms of HMPX**			
Yes	372 (77.8)	106 (22.2)	*P* = 0.016*
No	124 (87.3)	18 (12.7)	χ2 = 9.894
**Blisters on the skin are one of the symptoms or signs of human monkeypox**			
Yes	364 (78.4)	100 (21.6)	*P* = 0037*
No	132 (84.6)	24 (15.4)	χ2 = 12.56
**Antibiotics are used in the treatment of HMPX**			
Yes	194 (72.4)	74 (27.6)	*P* < 0.001*
No	302 (85.8)	50 (14.2)	χ2 = 19.064
**Diarrhea is one of the symptoms or signs of HMPX**			
Yes	46 (65.7)	24 (34.3)	*P* = 0.248
No	450 (81.8)	100 (18.2)	χ2 = 6.689
**Vaccination is available to prevent human monkeypox**			
Yes	274 (79.2)	72 (20.8)	*P* = 0.025*
No	222 (81.1)	52 (18.9)	χ2 = 19.794

*p < 0.05

### Overall knowledge level across subgroups

Table 4 showed that age and profession were the only significant factors associated with better HMPX knowledge.

**Table 4:** Factors associated with human monkeypox (HMPX) knowledge in the whole study sample

**HCWs’ demographic characteristics**	**Knowledge**			**ARR (95% Cl)**	***P* value**
	**Good *N* (%)**	**Moderate *N* (%)**	**Poor *N* (%)**		
**Age (years)**					
30	518 (83.5)	46 (7.5)	30 (4.8)	11.15 (0.29–1.66)	0.041*
≥ 30	16 (2.6)	6 (1)	4 (0.6)		
**Gender**					
Female	354 (57.1)	114 (18.4)	66 (10.6)	1.68 (0.44–1.83)	0.441
Male	40 (6.5)	26 (4.2)	20 (3.2)		
**Education**					
University and below	338 (54.5)	56 (9.1)	36 (5.8)	11.33 (0.37–0.73)	0.378
Postgraduate	156 (25.1)	26 (4.2)	8 (1.3)		
**Professions**					
Physician	406 (65.5)	54 (8.7)	36 (5.8)	10.57 (0.32–0.99)	0.023*
Other health care worker	66 (10.6)	42 (6.8)	16 (2.6)		
**Type of workplace**					
Community health care	216 (34.8)	98 (15.8)	92 (14.8)	10.29 (0.42–1.75)	0.621
General hospital	84 (13.5)	20 (3.2)	20 (3.2)		0.415
Private hospital	40 (6.6)	32 (5.2)	18 (2.9)	0.87 (0.37–0.93)	
**Have you ever received information about HMPX during education?**					
Yes	56 (9)	34 (5.5)	6 (1)	11.17 (0.35–1.11)	0.453
No	400 (64.5)	82 (13.2)	42 (6.8)		
**Have you ever heard about human monkeypox before**					
Yes	42 (6.8)	20 (3.2)	12 (1.9)	11.55 (0.37–1.86)	0.682
No	396 (63.9)	94 (15.2)	56 (9)		

*p < 0.05

### Beliefs items

Approximately half of the participants thought that viruses are biological weapons and I am skeptical about the official explanation regarding the cause of virus emergence. In addition, 60.3% of the study sample reported not confident information about the viruses from scientific specialists. On the other hand more than half of the participants do not believe that the virus is a hoax. Most viruses are artificial and the spread of viruses is a deliberate effort by a group of influential people to make money shown in Table 5.

**Table 5:** Beliefs items

**Beliefs items**	**Agree *N* (%)**	**Neutral *N* (%)**	**Disagree *N* (%)**
I am skeptical about the official explanation regarding the cause of virus emergence	354 (57.1)	110 (17.7)	156 (25.2)
The virus is a hoax	146 (23.5)	160 (25.9)	314 (50.6)
Most viruses are artificial	176 (28.4)	70 (11.3)	374 (60.3)
I do not confidence the information about viruses from scientific specialists	374 (60.3)	110 (17.8)	136 (21.9)
The transmission of viruses is a deliberate effort by a group of influential people to make money	206 (33.2)	100 (16.2)	314 (50.6)
The spread of viruses is a deliberate attempt by global companies to take control.	226 (36.5)	132 (21.2)	262 (42.3)
Lockdowns in response to emerging infection are aimed at mass surveillance and to destabilize the economy for financial gain	242 (39.0)	98 (15.8)	280 (45.2)
Lockdowns in response to emerging infections are aimed at mass surveillance and controlling every aspect of our lives	194 (31.3)	90 (14.5)	336 (54.2)
Lockdown is a method to isolate, terrify and demoralize a society as a whole to reshape society to fit specific interests	138 (22.3)	88 (14.2)	394 (63.5)
Viruses are biological weapons manufactured by the superpowers to take universal control	362 (58.4)	92 (14.8)	166 (26.8)

A greater mean score indicated a greater belief was found among females (*p* = 0.043). Physicians’ mean scores were lower than those of the other HCWs (*p* = 0.027). Table 6 shows that respondents under 30 had a higher mean score than respondents 30 or older (*p* = 0.031).

**Table 6:** Factors associated with human monkeypox (HMPX) beliefs in the whole study sample

**HCWs’ demographic characteristics**	**Beliefs**			**ARR (95% Cl)**	***P* value**
	**Agree *N* (%)**	**Neutral *N* (%)**	**Disagree *N* (%)**		
**Age (years)**					
30	416 (67.1)	102 (16.4)	76 (12.3)	10.51 (0.27–0.99)	0.031*
≥ 30	12 (1.9)	8 (1.3)	6 (1)		
**Gender**					
Female	446 (71.9)	52(8.4)	36 (5.8)	0.68 (0.37–0.93)	0.043*
Male	40 (6.5)	36 (5.8)	10 (1.6)		
**Education**					
University and below	296 (47.7)	88 (14.2)	46 (7.4)	11.11 (0.74–1.73))	0.405
Postgraduate	122 (19.7)	48 (7.7)	20(3.3)		
**Professions**					
Physician	38 (6.1)	60 (9.7)	398 (64.3)	10.57 (0.32–0.97)	0.027*
Other health care worker	12 (1.9)	46 (7.4)	66 (10.6)		
**Type of workplace**					
Community health care	76 (12.3)	100 (16.1)	230 (37.1)	11.15 (0.72–1.85)	0.406
General Hospital	6 (1)	16 (2.6)	102 (16.6)		
Private hospital	12 (1.9)	36 (5.8)	42 (6.8)	1.12 (0.65–1.83)	0.515
**Have you ever received information about human monkeypox during education**					
Yes	10 (1.6)	36 (5.8)	50 (8.1)	10.77 (0.45–1.21)	0.354
No	64 (10.3)	88 (14.2)	372 (60)		
**Have you ever heard about human monkeypox before**					
Yes	4 (0.6)	16 (2.6)	54 (8.7)	10.96 (0.47–1.96)	0.851
No	56 (9)	84 (13.5)	406 (65.5)		

*p < 0.05

## Discussion

The current study found that HCWs have knowledge gaps about the HMPX infection. This finding was observed in spite of extensive media coverage of the issue and the prompt and timely publication of literature covering almost all aspects of the condition [30, 31].

Therefore, consideration of HCWs’ knowledge to deal with potential threats of reemerging viruses is necessary for the development of efficient and well-organized response plans. In order to care for patients, help in control efforts, and deal with potential difficulties with stress and mental health problems typically experienced by HCWs in epidemic conditions, frontline HCWs need the correct direction [26].

The findings of our study were consistent with recent and more current research that revealed lacks in knowledge level of HMPX between physicians in Indonesia, health students in Jordan and Health Professionals in Kuwait [32].

In the current study, the level of awareness about monkeypox was higher than that reported for the general population in Saudi Arabia [33] as was to be expected.

Physicians demonstrated a higher degree of awareness about the condition despite the overall inadequate HMPX knowledge level that was described in this study. This outcome was consistent with that of a recent research reported among Jordanian HCWs [22]. The poorer knowledge of HMPX among other HCWs recommended more efforts to educate and train HCWs [34].

In this study, it was discovered that 31.3% of the study participants thought that “lockdowns in response to emerging infection are aimed at mass surveillance and to control every aspect of our lives,” as well as 58.4% who thought that “viruses are biological weapons manufactured by the superpowers to take global control.” According to Freeman et al. study, a significant percentage of people who held such beliefs were associated with medical mistrust and lower levels of compliance with COVID-19 pandemic control measures [21]. In our study, poorer HMPX knowledge was linked with higher acceptance of these Beliefs about emerging viral infection, which is similar to recent studies among HCWs in Jordan [4]. It is essential to emphasize that the cross-sectional study design cannot be used to assess the present direction of this link or the cause-effect relationship. The findings were also discussed in relation to Kuwaiti healthcare workers’ hesitation to get the COVID-19 vaccine and their vaccine-related conspiracies [22, 35].

This study also discovered that younger HCWs were likelier to have good knowledge than older workers. The younger HCWs have better access to information regarding MPX, which is mostly available online because they are more familiar with using the internet. Older doctors may also rely more on their personal experiences than information acquired from outside sources [36].

It is common for new physicians to work in community health centers, which are primary healthcare facilities and offer both curative and preventive services to the general public. Female sex and other HCWs are associated with higher Beliefs regarding virus emergence in this study. A similar pattern was observed by Sallam et al. in a recent survey among Students in Jordanian Health [7].

In this study, the most reported information about viruses was received by experts. This outcome is supported by data from recent research conducted in Turkey [37]. The contribution of scientists, physicians, and scientific journals emphasizes the importance of information sources in giving accurate knowledge, which may have a positive effect on health behavior.

Knowledge gaps about HMPX were found in this study’s analysis of human-to-human transmission. 36.8% of the participants correctly answered “human-to-human transmission of monkeypox occurs easily.” for the question. Recent studies using the same knowledge item have found similar results [10, 22, 38]. Prior to the outbreak in 2022, reports of human-to-human transmission of MPXV were made, and this is now becoming clear. However, it would be highlighted that the spread needs close contact not occur as frequently as diseases produced by respiratory viruses (such as SARS-CoV-2) [39]. Consequently, the importance of providing correct information about illness between HCWs cannot be ignored. As a result, this strategy between HCWs can help in educating the public and offering recommendations for patients, taking into account their significant role during this outbreak. This strategy should be motivated by the perfect knowledge required to be aware but not worried. A significant proportion of the participants in this study (31.5%) misidentified diarrhea as an HMPX symptom. For a prompt diagnosis of HMPX and the following application of control measures like contact tracing and isolation, a high index of suspicion is required. However, a lack of accurate information on the wide range of HMPX clinical symptoms could result in unnecessary diagnostic tests and the development of patient anxiety, wasting vital resources [40].

## Conclusion

A sample of HCWs had knowledge gaps about HMPX that were found in Egypt. Our findings revealed that low level of human monkeypox knowledge through sociodemographic, medical professional characteristics, workplace, as well as various levels of previous exposure to MPX information. Furthermore, younger physicians and those employed in community health centers appear to be more well-informed about MPX than older physicians and those working in private and general hospitals. There were numerous beliefs on new viral diseases. Future research should focus on the impact of the widespread adoption of conspiratorial thoughts on the response to the HMPX outbreak. These beliefs call for rapid, effective responses.

## Recommendations

We recommend the public and HCWs with less education to participate in health education programs. Continuous training of all HCWs on proper infection prevention measures.

## Limitations

It is important to acknowledge the study’s limitations. The cross-sectional design of the study limits the causality of the findings. The data collection method was via an online survey, another limitation that could carry a risk for non-response bias and may lead to different characteristics between the non-respondents and the respondents. The study researchers tried to maximize the sample size by forwarding the survey link to the different social media platforms and extending the data collection period to overcome the impact of these biases.

In Mansoura City (El Dakahlia Governorate) not all HCWs were represented in all hospital categories; some HCWs worked in private and general hospitals.

## Data Availability

On reasonable request, the corresponding author will provide the datasets created and used in the current study.
